# Other iatrogenic immunodeficiency-associated lymphoproliferative disorder presenting with multiple facial tumors

**DOI:** 10.1016/j.jdcr.2024.03.022

**Published:** 2024-04-21

**Authors:** Maya Kaga, Hirofumi Amano, Takayuki Kon, Sakiko Harada, Kanako Ogura

**Affiliations:** a Department of Dermatology and Allergy, Juntendo University Nerima Hospital, Tokyo, Japan; b Department of Rheumatology and Internal Medicine, Juntendo University Nerima Hospital, Tokyo, Japan; c Department of Hematology, Juntendo University Nerima Hospital, Tokyo, Japan; d Department of Pathology, Juntendo University Nerima Hospital, Tokyo, Japan

**Keywords:** Epstein-Bar Virus, Janus kinase inhibitor, lymphoproliferative disorder, methotrexate, other iatrogenic immunodeficiency-associated LPDs, tofacitinib

## Introduction

Iatrogenic immunodeficiency-associated lymphoproliferative disorders are characterized by lymphoid proliferation or lymphomas associated with iatrogenic immunodeficiencies.^1^ Other iatrogenic immunodeficiency-associated lymphoproliferative disorder (OIIA-LPD), a category delineated in the fourth edition of the World Health Organization of Hematolymphoid Tumors, includes methotrexate-related lymphoproliferative disorder as a severe complication in patients with rheumatoid arthritis (RA).^2^ Harada et al reported that 45.2% to 52.2% of MTX-related OIIA-LPD cases in patients with RA occurred in association with Epstein-Barr virus infection or reactivation.^3^ Cutaneous ulcers are the classic manifestation of Epstein-Barr virus-related OIIA-LPDs. Recently, therapeutic strategies using targeted synthetic or biological disease-modifying antirheumatic drugs (DMARDs), in addition to conventional synthetic DMARDs, including MTX, have become more common in patients with RA.^4^ These treatments improve the prognosis of RA. However, a history of treatment with Janus kinase (JAK) inhibitors may exacerbate the clinical aggressiveness of methotrexate (MTX)-related OIIA-LPDs in patients with RA.^4^ Here, we discuss a case of OIIA-LPD characterized by multiple facial tumors with an unusual clinical presentation.

## Case report

**Fig 1:**
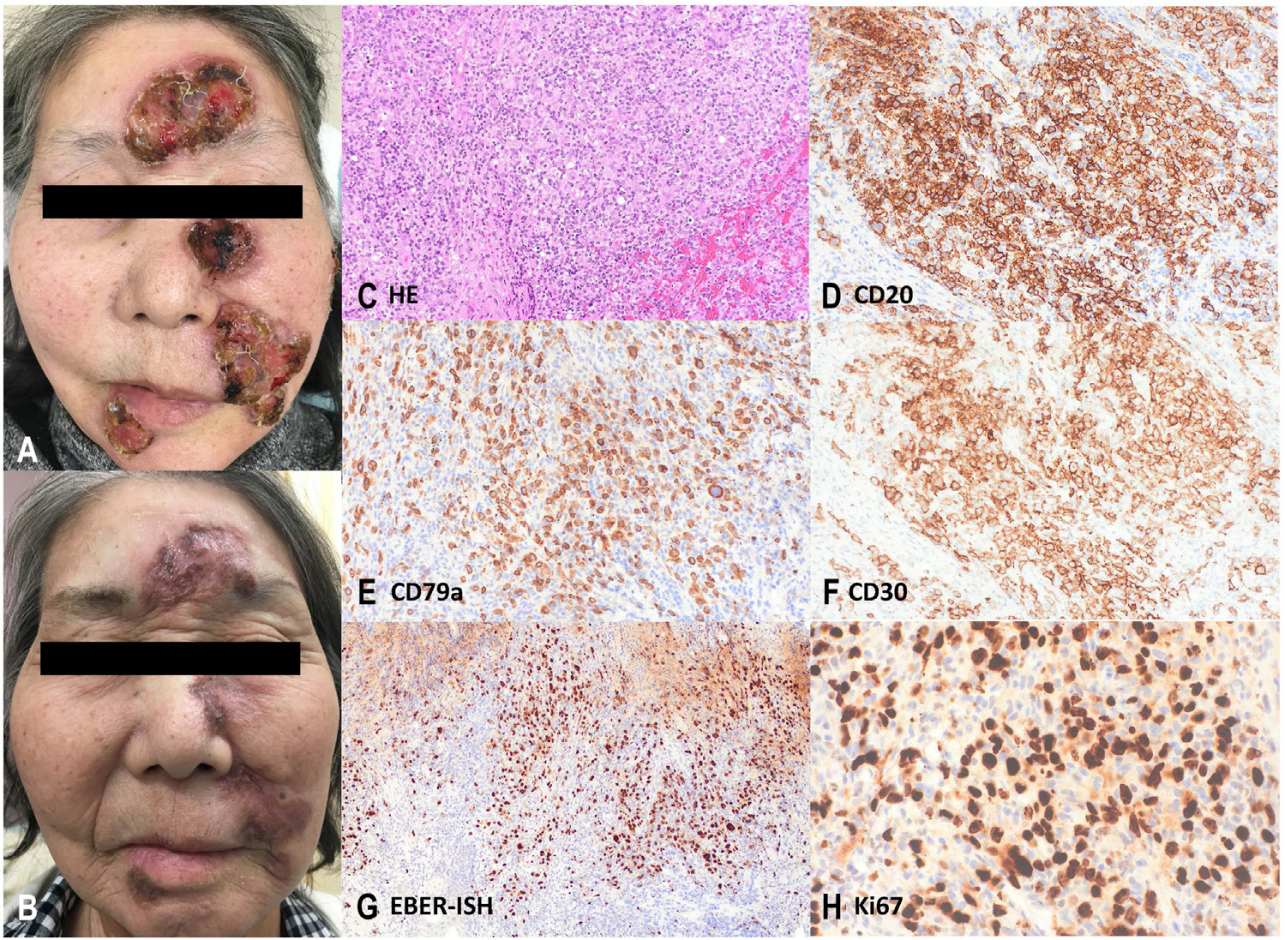
**A,** Skin tumors, partially ulcerated or granulated, of LPD on face. **B,** Postinflammatory pigmentation with mild itching was observed 2 months after MTX discontinuation and 1 month after Tofacitinib discontinuation. **C,** Histology of skin biopsy shows diffuse proliferation of lymphocytes with large nuclei including Hodgkin-like cells, in dermis and subcutaneous adipose tissue in Hematoxylin and eosin staining × 100. Immunohistochemistry; positive saining of CD20 (× 100), CD79a (× 100), CD30 (× 100), and EBER-ISH (**D-G**, × 100). Ki67 staining was observed in 85% of lymphocytes (**H**, × 200). *EBER-ISH*, Epstein-Bar Virus-Encoded Ribonucleic Acid In Situ Hybridization; *LPD*, lymphoproliferative disorder; *MTX*, methotrexate.

A 71-year-old woman presented with ulcerated tumors on her face that had increased in number and size over the past 5 months. Her medical history was significant for RA concomitant with polymyositis over the past 17 years. She had been treated with MTX 10 mg/week for 17 years since the onset of RA. Tofacitinib 5 mg/day, which inhibits JAK1 and JAK3, was added 3 years prior and enabled her to terminate systemic prednisolone, which she had initiated with 30 mg/day and taken for the previous 15 years. The patient presented to our department complaining of severe pruritus. Partially ulcerated and crusted tumors 2–5 cm in diameter were observed on the forehead, left cheek, philtrum, and chin (Fig 1, *A*). A skin biopsy revealed diffuse proliferation of atypical lymphocytes. Immunohistochemical analysis revealed positive staining for CD20, CD79a, CD30, and Epstein-Bar Virus-Encoded Ribonucleic Acid In Situ Hybridization (Fig 1, *C*-*G*). The patient was diagnosed with an iatrogenic immunodeficiency-associated LPD. The MTX treatment was immediately discontinued. Although the tumors seemed to stop progressing and the surfaces of the ulcerated tumors became dry, the size of the lesions remained unchanged. Consequently, tofacitinib was discontinued 1 month later, and iguratimod 25 mg/day was initiated simultaneously. These lesions resolved within a month after withdrawal of tofacitinib without chemotherapy (Fig 1, *B*). There were no signs of tumor relapse after 1 year of follow-up at our hospital.

## Discussion

IA-LPD, including LPD with primary immunodeficiency, HIV infection, posttransplantation, and OIIA-LPD, are grouped and discussed separately in the fourth edition of the World Health Organization of Hematolymphoid Tumors. As new types of immunodeficiency setting, such as immune modulatory agents and checkpoint inhibition therapies, have been recognized, IA-LPDs have been redefined as polymorphic lymphoproliferative disorders arising from immune deficiency/dysregulation in the fifth edition of the WHO classification of hematolymphoid tumors in 2022.^1^

Various clinical presentations of OIIA-LPDs were observed, including lymphadenopathy, skin tumors/ulcers, and organ tumors in the lungs, liver and spine. These cases are often diagnosed based on biopsy of lymph nodes or various organs/sites, such as the maxillary and mucocutaneous lesions involving the mandibular gingiva, eyelid, buccal mucosa, tongue, and sublingual area. Among the pathological subtypes of OIIA-LPD, diffuse large B-cell lymphoma a, polymorphic-type lymphoma, and classical Hodgkin’s lymphoma are the most frequently identified. Prior treatment with targeted synthetic or biological DMARDs did not influence the histopathological subtypes of MTX-related OIIA-LPD.^4^

According to a clinical analysis of 21 cases of MTX-induced OIIA-LPD in Japan, the mean duration of MTX use was 71.1 months.^5^ The rate of LPD regression after MTX withdrawal was 86.2% in 302 cases in Japan.^2^ Three prognostic subtypes of LPD have been proposed: group 1, regressive LPD without relapse/regrowth events; group 2, regressive LPD with relapse/regrowth; and group 3, persistent LPD after termination of immunosuppressive drugs.^6^ Patients in Group 1, with estimated monitored years of 1-13 years in their report, probably need to be closely monitored within 2-3 years of LPD regression^2^ before they are deemed cured. Although the mechanisms of action of JAK inhibitors in OIIA-LPD are unclear, JAK 1/2 inhibitors can contribute to the development of B-cell lymphoma by reducing T cell function and the frequency of circulating T cells.^4^

The differential diagnoses in our case included pyoderma gangrenosum or fungal/mycobacterial infection with underlying conditions of RA, polymyositis, and immunosuppressive treatment. The patient experienced spontaneous regression of the LPD resulting from a quick decision to discontinue both MTX and JAK inhibitors. Although the patient did not require chemotherapy, careful observation is essential to identify signs of possible relapse or regrowth of LPD.

## Conflict of interest

None disclosed.
